# CLINICAL EFFICACY OF A DIGITAL COGNITIVE ASSESSMENTS IN DETECTING COGNITIVE IMPAIRMENTS: A POOLED ANALYSIS

**DOI:** 10.1093/geroni/igae098.3936

**Published:** 2024-12-31

**Authors:** Duong Huynh, Mary Patterson, Bin Huang

**Affiliations:** BrainCheck, Austin, Texas, United States; BrainCheck, Austin, Texas, United States; BrainCheck, Austin, Texas, United States

## Abstract

Dementia has become a public health concern worldwide with significant social and economic impacts. Early detection of dementia, which involves diagnosis of mild cognitive impairment (MCI), a possible prestage of dementia, is crucial for timely interventions, care planning, and reducing healthcare costs. To promote proactive screening, it is important to equip primary care providers with assessment tools that are not only validated and reliable but also easy-to-administer and seamlessly integrated into their routine workflows. BrainCheck’s cognitive assessment tool (BC-Assess), with its demonstrated high diagnostic efficacy, usability, and feasibility of implementation, offers a promising solution. This study aimed to re-evaluate the efficacy of BC-Assess in identifying MCI and dementia based on a pooled dataset. The dataset included BC-Assess data of 193 individuals (age mean=73.7; SD=11.2), comprising those with normal cognition (NC; N=48) and those diagnosed with MCI (N=53) or dementia (N=92), acquired from two previous studies and from BrainCheck’s clinical partners. Each individual’s cognitive status was determined based on results from provider clinical diagnoses (N=158; 82%) or from the Montreal Cognitive Assessment (N=35; 18%). Significant differences in BC-Assess overall scores were found between the three groups (p<.001). ROC analysis shows that BC-Assess can achieve high sensitivity (>=85%) and specificity (>=81%) in distinguishing MCI and dementia patients from those with normal cognition, comparable with performance level obtained previously from a single data source. These results demonstrate the ability of BC-Assess to hold its diagnostic efficacy over combined data collected from different settings.

